# Effects of Marine *n*-3 Polyunsaturated Fatty Acids on Heart Rate Variability and Heart Rate in Patients on Chronic Dialysis: A Randomized Controlled Trial

**DOI:** 10.3390/nu10091313

**Published:** 2018-09-17

**Authors:** Jesper M. Rantanen, Sam Riahi, Martin B. Johansen, Erik B. Schmidt, Jeppe H. Christensen

**Affiliations:** 1 Department of Nephrology, Aalborg University Hospital, 9000 Aalborg, Denmark; jeppe.hagstrup.christensen@rn.dk; 2 Department of Clinical Medicine, Aalborg University, 9000 Aalborg, Denmark; sar@rn.dk (S.R.); ebs@rn.dk (E.B.S.); 3 Department of Cardiology, Aalborg University Hospital, 9000 Aalborg, Denmark; 4 Unit of Clinical Biostatistics, Aalborg University Hospital, 9000 Aalborg, Denmark; martin.johansen@rn.dk

**Keywords:** end-stage renal disease, dialysis, heart rate variability, heart rate, sudden cardiac death, *n*-3 polyunsaturated fatty acids, fish oils

## Abstract

Marine *n*-3 polyunsaturated fatty acids (PUFA) may improve autonomic dysfunction, as indicated by an increase in heart rate variability (HRV) and reduce the risk of sudden cardiac death. Hence, the aim of this study was to investigate the effects of marine *n*-3 PUFA on 24-h HRV in patients on chronic dialysis, who have a high risk of sudden cardiac death. Between June 2014 and March 2016, 112 patients on chronic dialysis from Denmark were allocated to a daily supplement of 2 g marine *n*-3 PUFA or control for three months in a randomized, double-blinded, controlled trial. A 48-h Holter monitoring was performed and mean 24-h HRV indices for the two days were available in 85 patients. The mean age was 62.3 years (SD: 14.3) and median dialysis vintage was 1.7 years (IQR: 0.5, 6.4). Within-group and between-group changes in outcome were evaluated by a paired and two sample *t*-test, respectively. Marine *n-*3 PUFA did not change the primary endpoint SDNN (SD of all RR-intervals) reflecting overall HRV, but other HRV indices increased and the mean RR-interval increased significantly, corresponding to a decrease in heart rate by 2.5 beats per minute (*p* = 0.04). In conclusion, marine *n*-3 PUFA did not change SDNN, but the mean heart rate was significantly reduced and changes in other HRV-indices were also observed, indicating an increase in vagal modulation that might be protective against malignant ventricular arrhythmias.

## 1. Introduction

Cardiovascular disease (CVD) is the leading cause of death and sudden cardiac death (SCD) accounts for approximately 30% of total deaths in patients with end-stage renal disease (ESRD) in the Western world [1,2]. SCD is frequently caused by malignant ventricular arrhythmias [3], but the reasons for the substantial increased risk of SCD in patients with ESRD are not fully understood. A combination of risk factors that are associated with ESRD and dialysis treatment may be part of the explanation. These include left ventricular hypertrophy, myocardial fibrosis, heart failure, volume overload, electrolyte abnormalities, changes in QT-dispersion, QT-prolonging medication, volume and electrolyte shifts during dialysis, and autonomic dysfunction [4]. Cardiac autonomic dysfunction, as characterised by excessive sympathetic activity and/or inadequate parasympathetic tone, is often seen in patients on chronic dialysis [5], which increases the risk of intradialytic hypotension [6] and predisposes to the development of malignant ventricular arrhythmias [7,8,9]. Cardiac autonomic dysfunction can be assessed by heart rate variability (HRV), which is an analysis of beat-to-beat variation of the heart rate (HR) [10]. An attenuated HRV is a strong predictor of mortality across a wide range of patient populations, including patients on chronic dialysis [9,11,12,13,14].

The marine *n*-3 polyunsaturated fatty acids (PUFA), eicosapentaenoic acid (EPA), and docosahexaenoic acid (DHA) have several effects that may reduce the risk of CVD. Some studies thus have reported a reduced cardiovascular mortality and a reduced risk of SCD [15,16], which might be due to an antiarrhythmic effect that is caused by a direct influence on the cardiomyocytes or indirectly by improving autonomic modulation of the heart shown by an increase in HRV [17,18]. Patients on chronic dialysis in the Western world have a low intake of marine *n*-3 PUFA with low levels in the blood [19,20]. It has been suggested that this may be a part of the explanation for a considerably higher risk of SCD in dialysis patients in the Western world when compared to in Japan, where the average marine *n*-3 PUFA intake is substantially higher [21]. Whether marine *n*-3 PUFA improve cardiac autonomic tone in patients on chronic dialysis is, however, uncertain, but we hypothesized that a daily supplement with marine *n*-3 PUFA would improve 24-h HRV and lower the mean HR in these patients.

## 2. Materials and Methods

### 2.1. Study Participants

The study was conducted between June 2014 and March 2016 at three hospital based dialysis units that were managed by Aalborg University Hospital treating all patients in the northern part of Denmark. Inclusion criteria were dialysis treatment for >3 months and age > 18 years. Exclusion criteria were pregnancy, expected life expectancy < 3 months, inability to give informed consent, known allergy to contents of the supplemental capsules, and expected inability to comply with the study protocol. All of the participants provided written informed consent and the protocol was approved by the regional committee on health research ethics and was in accordance with the Declaration of Helsinki. The trial was monitored by an external monitor to ensure compliance to the principles that were set forth in the guidelines for good clinical practice.

### 2.2. Study Design

This study was an investigator initiated randomized, double-blinded, placebo-controlled trial (ClinicalTrials.gov, unique identifier NCT02147977). Patients were randomized in a 1:1 ratio to receive 3 months daily supplement with 2 g *n*-3 PUFA (four capsules) containing a total of 1 g EPA + 1 g DHA or control capsules of identical appearance containing olive oil. A computer-generated permuted block randomization determined the allocation sequence. Patients, investigators and study personal were blinded to the allocation until the trial and all the analyses were completed. The patients were encouraged to maintain their usual fish intake during the study. Patients already taking *n*-3 PUFA supplements (*n* = 7) had at least eight weeks of washout prior to randomization. Compliance was assessed by counting remaining capsules that was returned at the end of the study. Patients were considered non-compliant if missing >15% of the prescribed capsules. Analysis of the content of *n*-3 PUFA in plasma phospholipids was done before and after supplementation as another indicator of adherence in the *n*-3 PUFA group. Adverse events were registered continuously and until two months after the last intake of the supplement. The following data were obtained at baseline and after three months supplementation: clinical assessment, detailed medical history, anthropometric data, blood pressure measurements (the mean of the last two of three readings after at least 5 min rest), venous blood samples, and a 48-h Holter monitoring.

### 2.3. Blood Samples

Non-fasting blood samples were drawn immediately before a dialysis session from the haemodialysis (HD) access in patients examined in the dialysis unit, and from venipuncture at a random time in home HD and peritoneal dialysis (PD) patients that were examined in the outpatient clinic. Routine haematological and biochemical analyses were done by standard methods at the central laboratory of the hospital. The fatty acid composition in plasma phospholipids were measured by gas chromatography (Varian 3900, Varian, Middleburg, The Netherlands), as previously described in detail [22]. Analyses of plasma phospholipid content of *n*-3 PUFA were done after the last patient finished the study and samples from each patient were done in the same run to avoid interassay variability.

### 2.4. Holter Monitoring and HRV Analysis

A 48-h Holter recording was obtained from each patient at baseline and after three months supplementation while using a two-channel digital recorder (Lifecard CF, Del Mar Reynolds Medical Limited, Hertford, UK) and analysed on each day separately with the results given as the mean value of the two days. In patients that were examined in the dialysis unit, the recording was initiated just before a dialysis session. Arrhythmia and time-domain HRV analyses were done using Pathfinder Digital 700 (Reynolds Medical Limited, Hertford, UK) by a semi-automated method with visual confirmation of all arrhythmias and exclusion of all non-sinus beats and artefacts from the HRV analysis. HRV analyses were only considered valid and used in the final analysis if at least 1152 min (80% of 24 h) of the recording were analysable with sinus rhythm. The following standard 24-h time-domain HRV indices were obtained: Mean RR-interval, the mean of all normal RR-intervals; SDNN, the standard deviation (SD) of all normal RR-intervals; SDNNi, the mean of the SD of all normal RR-intervals for all 5-min segments; SDANN, the SD of the mean of RR-intervals in successive 5-min segments; rMSSD, the square root of the mean of the sum of the squares of differences between adjacent RR-intervals; and, triangular index, total number of all RR-intervals divided by the height of the histogram of all RR-intervals measured in a discrete scale with bins of 7.8125 ms. Furthermore, using HRV tools (Reynolds Medical Limited, Hertford, UK), frequency-domain analysis was performed in 5-min segments averaged for each 24 h, obtaining the following indices: low frequency (LF, 0.04–0.15 Hz), high frequency (HF, 0.15–0.4 Hz), and LF/HF ratio.

### 2.5. Statistical Analyses

Categorical variables were presented as numbers and percentages. Continuous variables were expressed as means ± SD or medians with interquartile ranges (IQR) depending on the distribution of the data. The differences in the outcomes between baseline and three months were analysed within and between the two groups. Within-group changes were evaluated by paired *t*-test and data on frequency-domain indices was log-transformed due to positive skewed distributions. Two sample *t*-tests were used to compare the changes between groups from baseline to end of supplementation. All the analyses were done as intention-to-treat analysis. Power calculation was based on 24-h SDNN. We expected a SD of 30 ms and a minimal relevant difference of 15 ms. At a significance level of α = 0.05, power (1-β) = 80% and an expected drop out of 10% a sample size of *n* = 140 was estimated to be appropriate. The statistical package Stata 14 (StataCorp. 2015. College Station, TX, USA) was used for all statistical analyses and a two-tailed *p*-value of <0.05 was considered to be statistically significant.

## 3. Results

**Figure 1:**
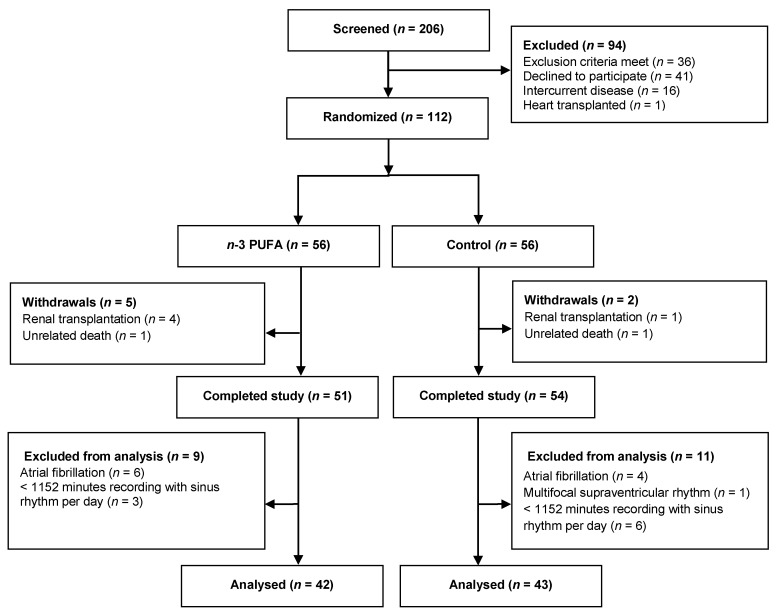
Patient enrolment, randomization and completion flow diagram. PUFA, polyunsaturated fatty acids; and, HRV, heart rate variability.

An overview of the patient flow is given in Figure 1. A total of 112 patients that were treated with in-center dialysis (77.7%), home HD (14.3%), or PD (8.0%) were enrolled and randomized evenly to each group. Before follow up, two patients died and five patients had a renal transplantation. The remaining 105 (94%) patients completed all the investigations in the trial, but unfortunately changes in the primary endpoint, HRV, was only available for analysis in 85 patients. HRV analysis requires sinus rhythm and most of the excluded patients (48%) were excluded due to atrial fibrillation on the recording and thus the excluded patients more often had a medical history of permanent or paroxysmal atrial fibrillation (63.0% vs. 16.5%, *p* < 0.001) and were older (69.0 ± 12.1 vs. 60.2 ± 14.3 years, *p* = 0.003) than the included patients.

Baseline characteristics of all randomized patients (*n* = 112) and patients that were included in the final HRV analyses (*n* = 85) were similar in the two supplement groups (Table 1). The only clinically relevant difference was a higher prevalence of myocardial infarction in the *n*-3 PUFA group.

### 3.1. Effects on HRV

Results from the HRV analyses are listed in Table 2. HRV indices in the two groups were similar before supplementation. The primary endpoint of 24-h SDNN and triangular index, which both reflect an estimate of overall HRV [10], increased significantly in the *n*-3 PUFA group, but there were no significant differences between the supplement groups. However, the change in SDNNi, which reflects the average variability due to cycles shorter than 5 min was significantly different between groups with an increase in the *n*-3 PUFA group and a decrease on the control group. Similarly, rMSSD and HF both reflecting vagal modulation [10] increased in the *n*-3 PUFA group and decreased in the control group, the group differences did not reach did not reach statistical significance. However, *n*-3 PUFA supplementation resulted in a significant increase in mean RR-interval between groups, corresponding to a decrease in HR of 2.5 beats per minute (bpm). Sensitivity analyses in patients without previous myocardial infarction (Table S1) and in in-center patients (Table S2) showed similar results as for the total group. Analysis for patients who adhered to the treatment (missing <15% prescribed capsules) also showed comparable results, but with a more pronounced HR lowering the effect of *n*-3 PUFA of 3.4 bpm (*p* = 0.01) (Table S3).

### 3.2. Effects on Plasma Lipids

Plasma total, high-density lipoprotein (HDL) or low-density lipoprotein (LDL) cholesterol levels did not change after supplement with 2 g marine *n*-3 PUFA, but plasma triglycerides decreased significantly by 0.33 mmol/L, according to a 17% reduction in plasma triglycerides in the *n*-3 PUFA group (Table 3).

### 3.3. Compliance

**Figure 2:**
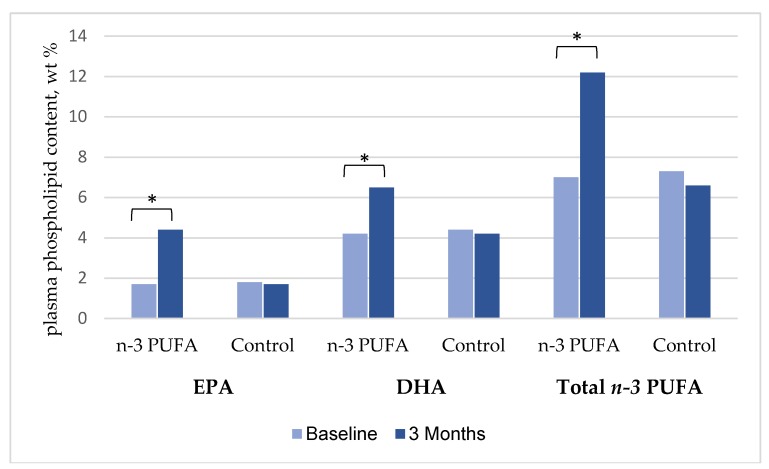
Plasma phospholipid content of EPA, DHA, and total *n*-3 PUFA (EPA + DHA + DPA) at baseline and after three months supplementation of 2 g *n*-3 PUFA. Significant changes are marked by * (*p* < 0.0001). EPA, eicosapentaenoic acid; DHA, docosahexaenoic acid; PUFA, polyunsaturated fatty acids; DPA, docosapentaenoic acid.

Compliance rates assessed by counts of returned capsules were similar with 76.5% in the *n*-3 PUFA group and 77.8% in the control group being compliant (*p* = 0.87). Consistent with the group assignments the mean plasma phospholipid content of DHA, EPA, and total *n*-3 PUFA increased significantly after three months of supplementation in the n-3 PUFA group, while no change was observed in the control group (Figure 2). Fish consumption evaluated by a questionnaire was similar at baseline and was found to be unchanged during the study period in both treatment groups (Table S4).

### 3.4. Tolerability and Adverse Events

Adverse events of any kind were observed in 70% of the patients (Table 4), but adverse events considered to be related to the supplements were only seen in 13 patients (nine in the *n*-3 PUFA group and 4 in the control group (*p* = 0.16)) and these comprised the gastrointestinal symptoms of nausea, vomiting, belching, and diarrhea, of which none were considered serious. Six patients in both treatment groups discontinued the treatment due to difficulties swallowing the capsules (*n* = 3), gastrointestinal adverse reactions (*n* = 4), or intercurrent diseases (*n* = 5), but completed the follow-up investigations and were included in the intention-to-treat analysis if HRV analyses were available (three patients in the *n*-3 PUFA group and five patients in the control group). One patient died in the *n*-3 PUFA group and two patients died in the control group, and all three deaths were considered to be unrelated to the supplements.

## 4. Discussion

A daily supplement with 2 g of marine *n*-3 PUFA for 12 weeks did not change SDNN reflecting overall HRV, but SDNNi increased significantly and there was a trend towards an increase in rMSSD and HF between groups, suggesting an increase in vagal modulation of the heart by marine *n*-3 PUFA. Furthermore, the mean RR-interval increased significantly corresponding to a decrease in mean HR by 2.5 bpm after supplementation.

Marine *n*-3 PUFA may have cardio-protective and antiarrhythmic effects in populations without kidney disease where a number of large randomized controlled trials (RCT) have reported that *n*-3 PUFA reduces major cardiovascular events, although data are not entirely consistent [15,16]. However, the literature on marine *n*-3 PUFA and mortality in patients with ESRD is sparse. Cohort and case-control studies have shown that *n*-3 PUFA are associated with a lower mortality or odds of SCD in chronic dialysis patients [21,23,24,25,26]. In an RCT, Svensson et al. [27] reported no effect of supplementation with 1.7 g marine *n*-3 PUFA for two years on a composite primary endpoint of total cardiovascular events and death in 206 HD patients with known CVD. However, a significant reduction in the risk of myocardial infarction as a secondary endpoint was observed.

Two small RCTs have examined the effects of *n*-3 PUFA supplements on HRV in patients on chronic dialysis [28,29]. In a study by Christensen et al. [28], 29 dialysis patients were allocated to 5.2 g *n*-3 PUFA or control for 12 weeks. No effect on 24-h time-domain HRV was detected between supplement groups, but after supplementation, a strong correlation between granulocyte levels of *n*-3 PUFA and 24-h SDNN was observed. Svensson et al. [29] randomized 43 HD patients to 1.7 g marine *n*-3 PUFA or control for three months with no effect observed of *n*-3 PUFA on 24-h time-domain HRV. A recently published cross sectional study from our center also failed to show associations between plasma phospholipid content of marine *n*-3 PUFA and time- and frequency domain HRV in 135 dialysis patients [30]. The present study is the largest study investigating the effects of marine *n*-3 PUFA supplements on autonomic function in patients on chronic dialysis. As ESRD patients, in general, our patients had an attenuated 24-h HRV at baseline [5,28,31]. SDNN did not change after supplementation with marine *n*-3 PUFA, but a significant effect on SDNNi and a borderline significant increase in rMSSD and HF was observed, indicating an improvement in vagal modulation. The changes in rMSSD and HF were partly due to a non-significant decrease in the control group, which might be due to worsening of vagal modulation as the dialysis vintage increases in the control group. The sample size was small and the findings could be by chance (type I error). Furthermore, several factors may have resulted in less effect of marine *n*-3 PUFA supplements in this study. A high number of the patients suffer from multiple comorbidities including diabetes and CVD that attenuate HRV. The use of medication that affect HRV [18,32] was also high. For instance, about two-thirds of the patients received a beta blocker and about half of the patients an angiotensin converting enzyme inhibitor or an angiotensin receptor blocker. The pronounced autonomic dysfunction at baseline may also have reduced the effect of marine *n*-3 PUFA on the outcomes [33].

The effects of marine *n*-3 PUFA supplementation on HRV in other populations have also shown inconsistent results. In high-risk patients with coronary heart disease or heart failure, some trials have shown a beneficial effect [34,35,36,37,38,39], whereas others showed no effect [40,41] on HRV. In a meta-analysis of 15 RCTs on various populations [42], time-domain HRV was not significantly influenced by marine *n*-3 PUFA supplementation, but frequency-domain HRV changed significantly with an increase in HF, supporting an effect of marine *n*-3 PUFA on vagal modulation also suggested by our data.

Mean RR-interval is the inverse of HR and not strictly a parameter of variability, but it is part of the basic description of HRV and a simple and a commonly used index of cardiac autonomic control [10]. One of the most consistent findings of marine *n*-3 PUFA is a HR lowering effect, which has been suggested to be part of the protective effect of *n*-3 PUFA against SCD [43], since an increased resting HR is associated with an increased mortality or risk of SCD in the general population [44,45,46]. A non-significant slowing of the HR was reported in previous studies in chronic dialysis patients [28,29], but we demonstrated a significant and substantial negative chronotropic effect (increase in mean RR-interval) at a similar level as in non-renal disease populations, which at a population level might have a clinically significant impact [43]. The negative chronotropic effect could be explained by an increase in vagal activity [43] or a direct effect on the intrinsic pacemaker rate of the sinus node [47]. Other benefits on cardiovascular physiology, including improved ventricular and endothelial function and lowering of blood pressure, may also result in a reduced HR [43].

Changes in the lipid profile may also be part of the cardio-protective mechanism of *n*-3 PUFA [16]. In the present study, a significant reduction in plasma triglycerides and no effect on plasma levels of total cholesterol, LDL-cholesterol, or HDL-cholesterol were observed. These results are in line with previous trials in patients with ESRD, who are known to have an atherogenic lipid profile [48]. Blood samples were non-fasting, but non-fasting samples are considered to be valid and recommended for evaluating lipid profiles [49].

The study had several strengths. The RCT design was of major importance. We used a three months intervention period, which should be sufficient to reach a steady state of marine *n*-3 PUFA in cardiac tissue [50], but whether this was obtained in neural tissues is uncertain. We were also able to document compliance by marked increases in mean DHA and EPA content in the plasma phospholipids in the *n*-3 PUFA group. Furthermore, instead of short-term HRV, we used 24-h HRV, which has a high reproducibility and seems free of placebo effect and has been suggested as a good method of assessing intervention therapies [10]. We also measured the outcomes on two separate days and calculated the mean values reducing the risk of chance findings (type 1 error). Olive oil was used as control, since it is known to have no antiarrhythmic properties [51], and to our knowledge, do not affect HRV. However, the study was also subject to limitations. Firstly, our enrolment goal was not reached resulting in a reduced power to detect differences in the primary endpoint (type 2 error). Secondly, the eligibility criteria were wide, resulting in a heterogeneous study population complicating the interpretation of comparisons between supplement groups with risk of residual confounding. However, the heterogeneity reflects daily clinical practice. Thirdly, despite randomization, more patients had a previous myocardial infarction in the *n*-3 PUFA group, but sensitivity analysis, excluding patients with myocardial infarction, showed similar results as the total group, thus showing evidence against the potential bias. Finally, despite 94% of the patients completing the study, only 85 (76%) of the randomized patients were included in the final analysis. However, the exclusions seem even and for similar reasons in the two groups minimizing the risk of bias.

## 5. Conclusions

In conclusion, supplementation with 2 g marine *n*-3 PUFA for three months did not change SDNN, but other HRV-indices increased, indicating an improvement in vagal modulation of the heart. Importantly, marine *n*-3 PUFA also had a significant and substantial HR lowering effect. We need future larger RCTs confirming our findings and investigating whether they translate into a protective effect against malignant ventricular arrhythmias and SCD in this high-risk population.

## Figures and Tables

**Table 1:** Baseline characteristics of all randomized patients and patients included in the final HRV analyses.

Parameters	*n*-3 PUFA All (*n* = 56)	*n*-3 PUFA HRV (*n* = 42)	Control All (*n* = 56)	Control HRV (*n* = 43)
Age, years	64.2 ± 14.5	61.9 ± 14.8	60.5 ± 13.9	58.6 ± 38.8
Sex, male, *n* (%)	37 (66.1)	30 (71.4)	37 (66.1)	27 (62.8)
Ethnicity, Caucasian, *n* (%)	56 (100)	42 (100)	53 (94.6)	41 (95.4)
Mean weight, kg	79.6 ± 16.9	80.0 ± 16.9	78.5 ± 19.9	79.5 ± 21.4
Body Mass Index, (kg/m^2^)	27.3 ± 6.1	26.9 ± 5.2	26.9 ± 6.2	27.4 ± 6.4
Current smoking, *n* (%)	9 (16.1)	6 (14.3)	14 (25.0)	10 (23.3)
Former renal transplantation, *n* (%)	8 (14.3)	7 (16.7)	10 (17.9)	6 (14.0)
Dialysis vintage, years	1.0 (0.5; 5.9)	1.0 (0.5; 6.0)	2.2 (0.5; 8.4)	1.5 (0.4; 6.4)
Systolic blood pressure, mmHg	143.1 ± 24.3	144.3 ± 26.4	143.8 ± 24.5	146.1 ± 23.4
Diastolic blood pressure, mmHg	71.6 ± 12.3	74.4 ± 12.0	73.0 ± 12.4	74.8 ± 12.1
Dialysis modality, *n* (%)				
In-center dialysis	43 (76.8)	32 (76.2)	44 (78.6)	32 (74.4)
Home haemodialysis	7 (12.5)	6 (14.3)	9 (16.1)	8 (18.6)
Peritoneal dialysis	6 (10.7)	4 (9.5)	3 (5.3)	3 (7.0)
Comorbidities, *n* (%)				
Hypertension	51 (91.1)	38 (90.5)	52 (92.9)	40 (93.0)
Diabetes mellitus	20 (35.7)	14 (33.3)	18 (32.1)	15 (34.9)
Cerebrovascular disease ^1^	18 (32.1)	13 (31.0)	13 (23.2)	11 (25.6)
Peripheral vascular disease ^2^	13 (23.2)	9 (21.4)	12 (21.4)	5 (11.6)
Myocardial infarction	12 (21.4)	8 (19.1)	5 (8.9)	3 (7.0)
Atrial fibrillation ^3^	15 (26.8)	8 (19.1)	16 (28.6)	6 (14.0)
Concomitant medication, *n* (%)				
Beta blocker	37 (66.1)	25 (59.5)	35 (62.5)	27 (62.8)
ACE-I or ARB	26 (46.4)	20 (47.6)	26 (46.4)	22 (51.2)
Anticoagulants	14 (25.0)	8 (19.1)	11 (19.6)	5 (11.6)
Aspirin	28 (50)	19 (45.2)	26 (46.4)	19 (44.2)
Clopidogrel	5 (8.9)	3 (7.1)	3 (5.4)	2 (4.7)
Statin	25 (44.6)	16 (38.1)	21 (37.5)	18 (41.9)
Biochemistry				
C-reactive protein, mg/L	3.8 (2.1; 8.9)	3.5 (1.9; 8.2)	3.6 (1.8; 11.5)	2.9 (1.7; 11.0)
Albumin, mg/L	32.1 ± 3.3	32.1 ± 3.2	33.1 ± 3.4	32.7 ± 3.3
Haemoglobin, mmol/L	6.9 ± 0.9	7.0 ± 0.8	6.8 ± 0.9	6.7 ± 0.9
High sensitive troponin T, ng/L	57 (37; 82)	55 (35; 80)	54 (31; 90)	54 (31; 88)
Phosphate, mmol/L	1.60 ± 0.40	1.58 ± 0.42	1.60 ± 0.48	1.60 ± 0.49
Plasma phospholipid *n*-3 PUFA				
EPA, wt %	1.85 ± 1.08	1.75 ± 1.08	1.75 ± 1.06	1.76 ± 1.16
DHA, wt %	4.29 ± 1.30	4.31 ± 1.32	4.29 ± 1.32	4.34 ± 1.32
DPA, wt %	1.12 ± 0.25	1.14 ± 0.25	1.11 ± 0.21	1.09 ± 0.22
Total n-3 PUFA, wt %	7.26 ± 2.18	7.21 ± 2.24	7.15 ± 2.28	7.19 ± 2.39

Data are mean ± standard deviation, median (interquartile range) or number *n* (%). ^1^ Cerebrovascular disease: ischemic stroke, haemorrhagic stroke and/or transient ischemic attack. ^2^ Peripheral vascular disease: amputation due to ischemia or claudication. ^3^ Atrial fibrillation: paroxysmal or permanent. HRV, heart rate variability; ACE-I, Angiotensin converting enzyme inhibitor; ARB, angiotensin receptor blocker; EPA, eicosapentaenoic acid; wt %, weight percent; DHA, docosahexaenoic acid; DPA, docosapentaenoic acid, PUFA, polyunsaturated fatty acids.

**Table 2:** Heart rate variability at baseline and after three months supplementation characteristics of all randomized patients and patients included in the final analyses.

Parameters	*n-*3 PUFA (*n* = 42)			Control (*n* = 43)			Difference in Response ^1^	*p*-Value
	Before	After	*p*-Value	Before	After	*p*-Value		
Time-domain HRV								
SDNN (ms)	84.3 ± 24.1	89.3 ± 27.4	0.047	85.2 ± 39.2	88.0 ± 33.0	0.23	2.1 (−4.7; 8.9)	0.54
SDANN (ms)	77.1 ± 21.9	81.9 ± 26.2	0.07	77.1 ± 36.3	81.3 ± 30.6	0.08	0.6 (−6.5; 7.7)	0.87
SDNNi (ms)	27.5 ± 12.0	28.6 ± 12.3	0.17	29.5 ± 15.4	28.0 ± 13.9	0.09	2.6 (0.3; 4.9)	0.03
rMSSD (ms)	14.4 ± 6.9	14.9 ± 6.7	0.57	15.0 ± 8.9	13.4 ± 6.8	0.02	2.0 (−0.02; 4.1)	0.05
Triangular Index	20.4 ± 5.6	22.7 ± 5.6	0.01	23.4 ± 11.9	23.7 ± 9.6	0.73	1.9 (−0.5; 4.4)	0.12
Mean RR (ms)	816.6 ± 116.3	834.3 ± 111.8	0.06	815.2 ± 121.0	804.6 ± 110.7	0.23	28.2 (3.4; 53.0)	0.03
Mean heart rate (bpm)	75.1 ± 11.3	73.2 ± 9.6	0.04	75.3 ± 11.6	76.0 ± 10.3	0.43	−2.5 (−5.0; −0.1)	0.04
Frequency-domain HRV ^2^								
LF (ms ^2^)	4.55 ± 1.31	4.63 ± 1.32	0.27	4.44 ± 1.51	4.39 ± 1.50	0.46	0.14 (−0.07; 0.36)	0.19
HF (ms ^2^)	3.66 ± 0.96	3.73 ± 0.92	0.53	3.72 ± 1.15	3.56 ± 1.19	0.06	0.23 (−0.04; 0.49)	0.09
LF/HF ratio	0.88 ± 0.88	0.92 ± 0.87	0.64	0.75 ± 0.93	0.85 ± 0.83	0.50	−0.06 (−0.24; 0.13)	0.55

Data are mean ± standard deviation or ^1^ absolute number and 95% confidence interval. ^2^ All frequency-domain indices are log-transformed due to skewed data. PUFA, polyunsaturated fatty acids; HRV, heart rate variability; mean RR, the 24-h mean value of RR-intervals; SDNN, the 24-h standard deviation of normal intervals; SDANN, the standard deviation of the mean of RR-intervals in successive 5-min segments; SDNNi, the mean of the standard deviation of all normal RR-intervals for all 5-min segments; rMSSD, the square root of the mean of the sum of the squares of differences between adjacent intervals; LF, low frequency; HF, high frequency.

**Table 3:** Effects of *n*-3 PUFA on plasma lipids (*n* = 105).

Parameters	*n*-3 PUFA (*n* = 51)			Control (*n* = 54)			Difference in Response ^1^	*p*-Value
	Before	After	*p*-Value	Before	After	*p*-Value		
Total-cholesterol, mmol/L	4.30 ± 1.35	4.20 ± 1.16	0.43	4.33 ± 1.24	4.33 ± 1.33	1.00	−0.09 (−0.39; 0.21)	0.54
HDL-cholesterol, mmol/L	1.19 ± 0.38	1.20 ± 0.35	0.83	1.19 ± 0.46	1.21 ± 0.42	0.61	−0.01 (−0.10; 0.08)	0.83
LDL-cholesterol ^2^, mmol/L	2.35 ± 1.12	2.37 ± 1.08	0.84	2.37 ± 1.04	2.36 ± 1.09	0.91	0.03 (−0.22; 0.28)	0.82
Triglycerides, mmol/L	1.77 ± 1.06	1.47 ± 0.75	0.001	1.82 ± 1.42	1.86 ± 1.43	0.68	−0.33 (−0.57; −0.10)	0.006

Data are mean ± standard deviation or ^1^ absolute number and 95% confidence interval. ^2^ LDL cholesterol was calculated. PUFA, polyunsaturated fatty acids; HDL, High-density lipoprotein; LDL, low-density lipoprotein.

**Table 4:** Adverse events.

Parameters	Total	*n*-3 PUFA (*n* = 56)	Control (*n* = 56)	*p*-Value
Nausea & vomiting	8	4	4	1.00
Bloating & belching	6	4	2	0.40
Diarrhea	8	5	3	0.46
Other gastrointestinal	8	5	3	0.46
Gastrointestinal bleeding	1	1	0	0.32
Intracerebral haemorrhage	1	0	1	0.32
Other bleeding	5	3	2	0.65
Infections	40	23	17	0.24
Death ^1^	3	1	2	0.31
Other various	52	30	22	0.13
Adverse events of any kind	78	43	35	0.10

Values are numbers of patients.^1^ one death in the placebo group occurred 14 days after last intake of placebo.

## Supplementary Materials

The following are available online at http://www.mdpi.com/2072-6643/10/9/1313/s1, Table S1: Heart rate variability at baseline and after three months supplementation in patients without previous acute myocardial infarction, Table S2: Heart rate variability at baseline and after three months supplementation in patients on in-center dialysis, Table S3: Heart rate variability at baseline and after three months supplementation in patients, who took at least 85% of the prescribed capsules (as treated analysis), Table S4: Fish intake before and after supplementation.

## References

[1] Herzog C.A., Mangrum J.M., Passman R. (2008). Sudden cardiac death and dialysis patients. Semin. Dial..

[2] (2016). United States Renal Data System. https://www.usrds.org/2016/download/v2_c09_CVD_16.pdf.

[3] Waks J.W., Tereshchenko L.G., Parekh R.S. (2016). Electrocardiographic predictors of mortality and sudden cardiac death in patients with end stage renal disease on hemodialysis. J. Electrocardiol..

[4] Teta D. (2013). Fish oil for prevention of sudden death in hemodialysis patients?. Kidney Int..

[5] Hathaway D.K., Cashion A.K., Milstead E.J., Winsett R.P., Cowan P.A., Wicks M.N., Gaber A.O. (1998). Autonomic dysregulation in patients awaiting kidney transplantation. Am. J. Kidney Dis..

[6] Yamamoto K., Kobayashi N., Kutsuna T., Ishii A., Matsumoto T., Hara M., Aiba N., Tabata M., Takahira N., Masuda T. (2012). Excessive fall of blood pressure during maintenance hemodialysis in patients with chronic renal failure is induced by vascular malfunction and imbalance of autonomic nervous activity. Ther. Apher. Dial..

[7] La Rovere M.T., Bigger J.T.J., Marcus F.I., Mortara A., Schwartz P.J. (1998). Baroreflex sensitivity and heart-rate variability in prediction of total cardiac mortality after myocardial infarction. ATRAMI (Autonomic Tone and Reflexes After Myocardial Infarction) Investigators. Lancet.

[8] Kleiger R.E., Miller J.P., Bigger J.T.J., Moss A.J. (1987). Decreased heart rate variability and its association with increased mortality after acute myocardial infarction. Am. J. Cardiol..

[9] Nishimura M., Tokoro T., Nishida M., Hashimoto T., Kobayashi H., Yamazaki S., Imai R., Okino K., Iwamoto N., Takahashi H. (2010). Sympathetic overactivity and sudden cardiac death among hemodialysis patients with left ventricular hypertrophy. Int. J. Cardiol..

[10] (1996). Heart Rate Variability: Standards of measurement, physiological interpretation and clinical use. Task force of the European society of cardiology and the North American society of pacing and electrophysiology. Circulation.

[11] Oikawa K., Ishihara R., Maeda T., Yamaguchi K., Koike A., Kawaguchi H., Tabata Y., Murotani N., Itoh H. (2009). Prognostic value of heart rate variability in patients with renal failure on hemodialysis. Int. J. Cardiol..

[12] Hayano J., Takahashi H., Toriyama T., Mukai S., Okada A., Sakata S., Yamada A., Ohte N., Kawahara H. (1999). Prognostic value of heart rate variability during long-term follow-up in chronic haemodialysis patients with end-stage renal disease. Nephrol. Dial. Transplant..

[13] Suzuki M., Hiroshi T., Aoyama T., Tanaka M., Ishii H., Kisohara M., Iizuka N., Murohara T., Hayano J. (2012). nonlinear measures of heart rate variability and mortality risk in hemodialysis patients. Clin. J. Am. Soc. Nephrol..

[14] Badarau S., Siriopol D., Drugus D., Dumea R., Hogas S., Blaj M., Voroneanu L., Gramaticu A., Petris A., Burlacu A. (2015). Electrocardiogram abnormalities and heart rate variability in predicting mortality and cardiovascular events among hemodialyzed patients. Int. Urol. Nephrol..

[15] De Caterina R. (2011). *N*-3 fatty acids in cardiovascular disease. N. Engl. J. Med..

[16] Saravanan P., Davidson N.C., Schmidt E.B., Calder P.C. (2010). Cardiovascular effects of marine omega-3 fatty acids. Lancet.

[17] McLennan P.L., Abeywardena M.Y. (2005). Membrane basis for fish oil effects on the heart: Linking natural hibernators to prevention of human sudden cardiac death. J. Membr. Biol..

[18] Christensen J.H. (2003). *N*-3 fatty acids and the risk of sudden cardiac death. Emphasis on heart rate variability. Dan. Med. Bull..

[19] Friedman A.N., Yu Z., Tabbey R., Denski C., Tamez H., Wenger J., Thadhani R., Li Y., Watkins B.A. (2012). Low blood levels of long-chain *n*-3 polyunsaturated fatty acids in US hemodialysis patients: clinical implications. Am. J. Nephrol..

[20] Madsen T., Christensen J.H., Svensson M., Witt P.M., Toft E., Schmidt E.B. (2011). Marine *N*-3 Polyunsaturated fatty acids in patients with end-stage renal failure and in subjects without kidney disease: A comparative study. J. Ren. Nutr..

[21] Terashima Y., Hamazaki K., Itomura M., Tomita S., Kuroda M., Hirata H., Hamazaki T., Inadera H. (2014). Inverse association between docosahexaenoic acid and mortality in patients on hemodialysis during Over 10 Years. Hemodial. Int..

[22] Eide I.A., Jenssen T., Hartmann A., Diep L.M., Dahle D.O., Reisaeter A.V., Bjerve K.S., Christensen J.H., Schmidt E.B., Svensson M. (2015). The association between marine *n*-3 polyunsaturated fatty acid levels and survival after renal transplantation. Clin. J. Am. Soc. Nephrol..

[23] Hamazaki K., Terashima Y., Itomura M., Sawazaki S., Inagaki H., Kuroda M., Tomita S., Hirata H., Inadera H., Hamazaki T. (2011). Docosahexaenoic acid is an independent predictor of all-cause mortality in hemodialysis patients. Am. J. Nephrol..

[24] Inoue T., Okano K., Tsuruta Y., Tsuruta Y., Tsuchiya K., Akiba T., Nitta K. (2015). Eicosapentaenoic acid (EPA) decreases the all-cause mortality in hemodialysis patients. Intern. Med..

[25] Friedman A.N., Saha C., Watkins B.A. (2008). Feasibility study of erythrocyte long-chain omega-3 polyunsaturated fatty acid content and mortality risk in hemodialysis patients. J. Ren. Nutr..

[26] Friedman A.N., Yu Z., Tabbey R., Denski C., Tamez H., Wenger J., Thadhani R., Li Y., Watkins B.A. (2013). Inverse relationship between long-chain *n*-3 fatty acids and risk of sudden cardiac death in patients starting hemodialysis. Kidney Int..

[27] Svensson M., Schmidt E.B., Jorgensen K.A., Christensen J.H., OPACH Study Group (2006). *N*-3 fatty acids as secondary prevention against cardiovascular events in patients who undergo chronic hemodialysis: A randomized, placebo-controlled intervention trial. Clin. J. Am. Soc. Nephrol..

[28] Christensen J.H., Aaroe J., Knudsen N., Dideriksen K., Kornerup H.J., Dyerberg J., Schmidt E.B. (1998). Heart rate variability and *n*-3 fatty acids in patients with chronic renal failure—A pilot study. Clin. Nephrol..

[29] Svensson M., Schmidt E.B., Jorgensen K.A., Christensen J.H. (2007). The effect of *n*-3 fatty acids on heart rate variability in patients treated with chronic hemodialysis. J. Ren. Nutr..

[30] Rantanen J., Schmidt E., Riahi S., Lundbye-Christensen S., Christensen J. (2018). Marine *n*-3 PUFA, heart rate variability and ventricular arrhythmias in patients on chronic dialysis: A cross-sectional study. Br. J. Nutr..

[31] Longenecker J.C., Zubaid M., Johny K.V., Attia A.I., Ali J., Rashed W., Suresh C.G., Omar M. (2009). Association of low heart rate variability with atherosclerotic cardiovascular disease in hemodialysis patients. Med. Princ. Pract..

[32] Erkkilä A.T., Schwab U.S., de Mello V.D.F., Lappalainen T., Mussalo H., Lehto S., Kemi V., Lamberg-Allardt C., Uusitupa M.I.J. (2008). Effects of fatty and lean fish intake on blood pressure in subjects with coronary heart disease using multiple medications. Eur. J. Nutr..

[33] Santini V., Ciampittiello G., Gigli F., Bracaglia D., Baroni A., Cicconetti E., Verri C., Gambardella S., Frontoni S. (2007). QTc and autonomic neuropathy in diabetes: Effects of acute hyperglycaemia and *n*-3 PUFA. Nutr. Metab. Cardiovasc. Dis..

[34] Christensen J.H., Skou H.A., Fog L., Hansen V., Vesterlund T., Dyerberg J., Toft E., Schmidt E.B. (2001). Marine *n*-3 fatty acids, wine intake, and heart rate variability in patients referred for coronary angiography. Circulation.

[35] Christensen J.H., Gustenhoff P., Korup E., Aaroe J., Toft E., Moller J., Rasmussen K., Dyerberg J., Schmidt E.B. (1996). Effect of fish oil on heart rate variability in survivors of myocardial infarction: A double blind randomised controlled trial. BMJ.

[36] Villa B., Calabresi L., Chiesa G., Rise P., Galli C., Sirtori C.R. (2002). Omega-3 fatty acid ethyl esters increase heart rate variability in patients with coronary disease. Pharmacol. Res..

[37] O’Keefe J.H., Abuissa H., Sastre A., Steinhaus D.M., Harris W.S. (2006). Effects of omega-3 fatty acids on resting heart rate, heart rate recovery after exercise, and heart rate variability in men with healed myocardial infarctions and depressed ejection fractions. Am. J. Cardiol..

[38] La Rovere M.T., Staszewsky L., Barlera S., Maestri R., Mezzani A., Midi P., Marchioli R., Maggioni A.P., Tognoni G., Tavazzi L. (2013). *N*-3PUFA and holter-derived autonomic variables in patients with heart failure: Data from the Gruppo Italiano per lo studio Della Sopravvivenza Nell’Insufficienza Cardiaca (GISSI-HF) Holter Substudy. Heart Rhythm.

[39] Nodari S., Metra M., Milesi G., Manerba A., Cesana B.M., Gheorghiade M., Dei Cas L. (2009). The role of *n*-3 PUFAs in preventing the arrhythmic risk in patients with idiopathic dilated cardiomyopathy. Cardiovasc. Drugs Ther..

[40] Hamaad A., Kaeng Lee W., Lip G.Y., MacFadyen R.J. (2006). Oral omega *n*-3-PUFA therapy (omacor) has no impact on indices of heart rate variability in stable post myocardial infarction patients. Cardiovasc. Drugs Ther..

[41] Carney R.M., Freedland K.E., Stein P.K., Steinmeyer B.C., Harris W.S., Rubin E.H., Krone R.J., Rich M.W. (2010). Effect of omega-3 fatty acids on heart rate variability in depressed patients with coronary heart disease. Psychosom. Med..

[42] Xin W., Wei W., Li X.Y. (2013). Short-Term effects of fish-oil supplementation on heart rate variability in humans: A meta-analysis of randomized controlled trials. Am. J. Clin. Nutr..

[43] Mozaffarian D., Geelen A., Brouwer I.A., Geleijnse J.M., Zock P.L., Katan M.B. (2005). Effect of fish oil on heart rate in humans: A meta-analysis of randomized controlled trials. Circulation.

[44] Jouven X., Empana J.P., Escolano S., Buyck J.F., Tafflet M., Desnos M., Ducimetiere P. (2009). Relation of heart rate at rest and long-term (>20 years) death rate in initially healthy middle-aged men. Am. J. Cardiol..

[45] Sharashova E., Wilsgaard T., Mathiesen E.B., Lochen M.L., Njolstad I., Brenn T. (2016). Resting heart rate predicts incident myocardial infarction, atrial fibrillation, ischaemic stroke and death in the general population: The Tromso study. J. Epidemiol. Community Health.

[46] Wannamethee G., Shaper A.G., Macfarlane P.W., Walker M. (1995). Risk factors for sudden cardiac death in middle-aged British men. Circulation.

[47] Billman G.E., Harris W.S. (2011). Effect of dietary omega-3 fatty acids on the heart rate and the heart rate variability responses to myocardial ischemia or submaximal exercise. Am. J. Physiol. Heart Circ. Physiol..

[48] Xu T., Sun Y., Sun W., Yao L., Sun L., Liu L., Ma J., Wang L. (2016). Effect of omega-3 fatty acid supplementation on serum lipids and vascular inflammation in patients with end-stage renal disease: A meta-analysis. Sci. Rep..

[49] Nordestgaard B.G., Langsted A., Mora S., Kolovou G., Baum H., Bruckert E., Watts G.F., Sypniewska G., Wiklund O., Boren J. (2016). Fasting is not routinely required for determination of a lipid profile: Clinical and laboratory implications including flagging at desirable concentration cutpoints—A joint consensus statement from the European atherosclerosis society and European federation of clinical chemistry and laboratory medicine. Clin. Chem..

[50] Metcalf R.G., James M.J., Gibson R.A., Edwards J.R., Stubberfield J., Stuklis R., Roberts-Thomson K., Young G.D., Cleland L.G. (2007). Effects of fish-oil supplementation on myocardial fatty acids in humans. Am. J. Clin. Nutr..

[51] McLennan P.L. (1993). Relative Effects of dietary saturated, monounsaturated, and polyunsaturated fatty acids on cardiac arrhythmias in rats. Am. J. Clin. Nutr..

