# Supplementary material for: Effects of Marine n-3 Polyunsaturated Fatty Acids on Heart Rate Variability and Heart Rate in Patients on Chronic Dialysis: A Randomized Controlled Trial
Source: Nutrients. 2018 Sep 17;10(9):1313. doi: 10.3390/nu10091313 (PMC6163625; doi:10.3390/nu10091313)
Supplement: Supplementary file 1 [file nutrients-10-01313-s001.pdf]

nutrients

MDPI

Supplementary Material

This file contains the following supplementary tables:

- **Table S1.** Heart rate variability at baseline and after three months supplementation in patients without previous acute myocardial infarction.
- **Table S2.** Heart rate variability at baseline and after three months supplementation in patients on in-center dialysis.
- **Table S3.** Heart rate variability at baseline and after three months supplementation in patients, who took at least 85% of the prescribed capsules (as treated analysis).
- **Table S4.** Fish intake before and after supplementation.

Table S1. Heart rate variability at baseline and after three months supplementation in patients without previous acute myocardial infarction.

Parameters	*n*-3 PUFA (*n* = 27)			Control (*n* = 38)			Difference in response[1]	
	Before	After	*p*-Value	Before	After	*p*-Value		*p*-Value
Time-domain HRV								
SDNN (ms)	87.5 ± 22.7	90.7 ± 28.8	0.33	85.8 ± 39.5	88.2 ± 31.4	0.35	0.8 (-7.3; 8.9)	0.84
SDANN (ms)	79.2 ± 20.4	82.1 ± 26.6	0.42	77.9 ± 36.8	81.3 ± 28.7	0.17	-0.4 (-8.8; 8.0)	0.92
SDNNi (ms)	30.1 ± 13.0	30.2 ± 13.8	0.88	29.2 ± 15.4	28.3 ± 14.2	0.27	1.1 (-1.6; 3.8)	0.41
rMSSD (ms)	14.5 ± 6.9	14.0 ± 6.8	0.60	14.9 ± 9.1	13.5 ± 7.0	0.04	0.9 (-1.3; 3.2)	0.41
Triangular Index	21.3 ± 6.1	22.6 ± 8.9	0.22	23.6 ± 12.1	23.5 ± 9.4	0.091	1.5.(-1.5; 4.4)	0.33
Mean RR (ms)	793.8 ± 116.6	803.6 ± 103.3	0.46	819.4 ± 118.1	810.6 ± 112.2	0.26	18.7 (-10.0; 47.3)	0.20
Mean heart rate (bpm)	77.3 ± 12.1	75.9 ± 9.6	0.28	74.7 ± 10.8	75.4 ± 10.3	0.36	-2.1 (-5.0; 0.7)	0.14
Frequency-domain HRV[2]								
LF (ms^2)	4.86 ± 1.25	4.86 ± 1.38	0.99	4.47 ± 1.56	4.43 ± 1.53	0.54	0.05 (-0.19; 0.28)	0.70
HF (ms^2)	3.73 ± 0.97	3.67 ± 0.97	0.66	3.76 ± 1.15	3.59 ± 1.19	0.06	0.11 (-0.21; 0.42)	0.51
LF/HF ratio	1.14 ± 0.81	1.19 ± 0.82	0.56	0.75 ± 0.97	0.85 ± 0.86	0.09	-0.05 (-0.25; 0.16)	0.64

Data are mean ± standard deviation or [1]absolute number and 95% confidence interval.
[2]All frequency-domain indices are log-transformed due to skewed data.
PUFA, polyunsaturated fatty acids; HRV, heart rate variability; mean RR, the 24-hour mean value of RR-intervals; SDNN, the 24-hour standard deviation of normal intervals; SDANN, the standard deviation of the mean of RR-intervals in successive 5-minute segments; SDNNi, the mean of the standard deviation of all normal RR-intervals for all 5 minute segments; rMSSD, the square root of the mean of the sum of the squares of differences between adjacent intervals; LF, low frequency; HF, high frequency.

nutrients MDPI

Table S2. Heart rate variability at baseline and after three months supplementation in patients on in-center dialysis.

Parameters	*n*-3 PUFA (*n* = 32)			Control (*n* = 32)			Difference in response[1]	
	Before	After	*p*-Value	Before	After	*p*-Value		*p*-Value
Time-domain HRV								
SDNN (ms)	84.3 ± 23.7	87.9 ± 26.7	0.17	83.5 ± 40.3	86.8 ± 34.2	0.25	0.3 (-7.4; 8.0)	0.94
SDANN (ms)	77.4 ± 22.2	81.2 ± 26.2	0.17	75.8 ± 37.6	80.6 ± 32.6	0.10	-1.0 (-8.9; 6.8)	0.80
SDNNi (ms)	26.2 ± 9.1	26.7 ± 9.9	0.63	28.7 ± 15.6	26.5 ± 13.4	0.06	2.6 (0.3; 4.9)	0.08
rMSSD (ms)	14.1 ± 6.5	14.2 ± 6.3	0.91	15.0 ± 11.6	12.9 ± 10.5	0.01	2.2 (-0.29; 4.8)	0.08
Triangular Index	19.9 ± 4.7	21.8 ± 6.6	0.04	22.4 ±11.6	23.4 ± 9.8	0.33	1.0 (-1.6; 3.6)	0.45
Mean RR (ms)	839.9 ± 112.1	860.2 ± 109.7	0.06	836.9 ± 126.0	820.8 ± 111.5	0.12	36.4 (7.4; 65.3)	0.01
Mean heart rate (bpm)	72.8 ± 20.3	70.9 ± 9.0	0.054	73.4 ± 11.6	74.4 ± 9.9	0.32	-2.9 (-5.7; 0.2)	0.04
Frequency-domain HRV[2]								
LF (ms²)	4.35 ± 1.22	4.41 ± 1.21	0.51	4.35 ± 1.45	4.26 ± 1.44	0.30	0.16 (-0.10; 0.41)	0.23
HF (ms²)	3.61 ± 0.87	3.62 ± 0.86	0.95	3.64 ± 1.17	3.42 ± 1.16	0.04	0.01 (-0.08; 0.53)	0.14
LF/HF ratio	0.75 ± 0.84	0.80 ± 0.81	0.53	0.75 ± 0.92	0.85 ± 0.84	0.15	-0.04 (-0.27; 0.18)	0.70

Data are mean ± standard deviation or [1]absolute number and 95% confidence interval.
[2]All frequency-domain indices are log-transformed due to skewed data.
PUFA, polyunsaturated fatty acids; HRV, heart rate variability; mean RR, the 24-hour mean value of RR-intervals; SDNN, the 24-hour standard deviation of normal intervals; SDANN, the standard deviation of the mean of RR-intervals in successive 5-minute segments; SDNNi, the mean of the standard deviation of all normal RR-intervals for all 5 minute segments; rMSSD, the square root of the mean of the sum of the squares of differences between adjacent intervals; LF, low frequency; HF, high frequency.

nutrients

MDPI

Table S3. Heart rate variability at baseline and after three months supplementation in patients, who took at least 85% of the prescribed capsules (as treated analysis).

Parameters	*n*-3 PUFA (*n* = 34)			Control (*n* = 34)			Difference in response[1]	
	Before	After	*p*-Value	Before	After	*p*-Value		*p*-Value
Time-domain HRV								
SDNN (ms)	83.8 ± 24.6	88.1 ± 25.9	0.08	94.9 ± 37.8	96.9 ± 30.7	0.49	2.3 (-5.3; 9.8)	0.55
SDANN (ms)	76.1 ± 22.0	80.3 ± 25.1	0.11	85.7 ± 35.4	89.1 ± 29.0	0.26	0.8 (-7.0; 8.6)	0.84
SDNNi (ms)	28.2 ± 12.0	29.4 ± 11.9	0.23	33.2 ± 15.0	31.8 ± 13.0	0.17	2.6 (-0.2; 5.5)	0.07
rMSSD (ms)	15.3 ± 7.2	15.7 ± 6.7	0.67	16.5 ± 9.4	14.9 ± 6.8	0.05	2.0 (-0.47; 4.5)	0.11
Triangular Index	20.5 ± 5.7	22.7 ± 7.0	0.02	26.2 ± 11.7	26.4 ± 8.9	0.82	1.9 (-0.9; 4.7)	0.17
Mean RR (ms)	816.7 ± 122.1	839.3 ± 119.1	0.01	849.1 ± 105.4	836.1 ± 97.8	0.22	35.6 (8.7; 62.6)	0.01
Mean heart rate (bpm)	75.2 ±11.9	72.9 ± 10.2	0.02	71.7 ± 8.6	72.7 ± 8.0	0.30	-3.3 (-5.0; 0.1)	0.01
Frequency-domain HRV[2]								
LF (ms²)	4.65 ± 1.22	4.74 ± 1.20	0.34	4.89 ± 1.19	4.88 ± 1.11	0.85	0.1 (-0.13; 0.34)	0.39
HF (ms²)	3.76 ± 0.97	3.83 ± 0.88	0.57	3.98 ± 1.11	3.88 ± 1.05	0.26	0.16 (-0.12; 0.46)	0.25
LF/HF ratio	0.89 ± 0.88	0.92 ± 0.82	0.69	0.94 ± 0.81	1.01 ± 0.72	0.25	-0.03 (-0.25; 0.18)	0.76

Data are mean ± standard deviation or [1]absolute number and 95% confidence interval.
[2]All frequency-domain indices are log-transformed due to skewed data.
PUFA, polyunsaturated fatty acids; HRV, heart rate variability; mean RR, the 24-hour mean value of RR-intervals; SDNN, the 24-hour standard deviation of normal intervals; SDANN, the standard deviation of the mean of RR-intervals in successive 5-minute segments; SDNNi, the mean of the standard deviation of all normal RR-intervals for all 5 minute segments; rMSSD, the square root of the mean of the sum of the squares of differences between adjacent intervals; LF, low frequency; HF, high frequency.

nutrients

MDPI

Table S4. Fish intake before and after supplementation.

Parameter	*n*-3 PUFA (*n* = 42)			Control (*n* = 43)			Difference in response[1]	
	Before	After	*p*-Value	Before	After	*p*-Value		*p*-Value
Fish score [2]	6.8 ± 2.6	6.8 ± 2.6	1.0	7.0 ± 2.4	6.7 ± 2.6	0.39	0.2 (-0.4; 0.9)	0.52

Data are mean ± standard deviation or [1]absolute number and 95% confidence interval.
[2]Fish intake assessed by a questionnaire with two questions: 1. How often do you eat fish for lunch? and 2. How often do you eat fish for dinner? A score was given according to the following: never eating fish=1; eating fish once a month=2, eating fish 2-3 times a month=3´, eating fish once a week=4, eating fish 2-3 times a week=5 and eating fish every day=6. Hence, the total fish score for lunch and dinner could range from 2-12.
